# An EM algorithm for mapping segregation distortion loci

**DOI:** 10.1186/1471-2156-8-82

**Published:** 2007-11-29

**Authors:** Chengsong Zhu, Yuan-Ming Zhang

**Affiliations:** 1 Section on Statistical Genomics, State Key Laboratory of Crop Genetics and Germplasm Enhancement/National Center for Soybean Improvement, Nanjing Agricultural University, Nanjing 210095, China

## Abstract

**Background:**

Chromosomal region that causes distorted segregation ratios is referred to as segregation distortion locus (SDL). The distortion is caused either by differential representation of SDL genotypes in gametes before fertilization or by viability differences of SDL genotypes after fertilization but before genotype scoring. In both cases, observable phenotypes are distorted for marker loci in the chromosomal region close to the SDL. Under the quantitative genetics model for viability selection by proposing a continuous liability controlling the viability of individual, a simplex algorithm has been used to search for the solution in SDL mapping. However, they did not consider the effects of SDL on the construction of linkage maps.

**Results:**

We proposed a multipoint maximum-likelihood method to estimate the position and the effects of SDL under the liability model together with both selection coefficients of marker genotypes and recombination fractions. The method was implemented via an expectation and maximization (EM) algorithm. The superiority of the method proposed under the liability model over the previous methods was verified by a series of Monte Carlo simulation experiments, together with a working example derived from the MAPMAKER/QTL software.

**Conclusion:**

Our results suggested that the new method can serve as a powerful alternative to existing methods for SDL mapping. Under the liability model, the new method can simultaneously estimate the position and the effects of SDL as well as the recombinant fractions between adjacent markers, and also be used to probe into the genetic mechanism for the bias of uncorrected map distance and to elucidate the relationship between the viability selection and genetic linkage.

## Background

In a segregation population derived from a cross between two inbred lines, some molecular markers often show distorted segregation ratios from Mendelian expectations [1-3]. The distortion is frequently related to gamete gene, sterile gene and chromosome translocation [4]. So the detection of the gene or locus, known as segregation distortion locus (SDL) mapping, is warranted. However, the challenge encountered in SDL mapping is mainly caused by the unavailability of phenotypic data for the underlying trait. In fact, molecular markers linked to the SDL frequently show segregation distortion and the degree of distortion depends on the size and the position of SDL. Therefore, it is possible to detect SDL by means of the distortion.

Mapping SDL is usually studied at the population level by examining the change of gene (or genotypic) frequencies of markers [5]. In the past a single marker was often used to detect the linkage between the marker and SDL [6,7]. Its shortcomings are very similar to those of single-marker approaches in quantitative trait loci (QTL) mapping [8]. Since the introduction of interval mapping of QTL [9], Hedrick and Muona [10] developed a flanking-marker analysis to estimate the fitness parameters for a viability locus. The model of Hedrick and Muona [10] is actually a complete recessive model. Mitchell-Olds [11] detected one putative viability locus at a time and then scanned the entire genome for every putative position to provide a test statistic profile for the detection of SDL. However, his model only test and estimate the degree of dominance. Luo and Xu [12] extended the maximum-likelihood (ML) method to estimate degree of dominance and selection coefficients using an outbred full-sib family as an example. Wang et al. [13] developed a multipoint ML method to estimate the position and the genotypic frequencies of SDL in an F_2 _population. However, the efficacies of the methods mentioned above have been seldom addressed in simulation studies. Recently, Luo et al. [14] developed a quantitative genetics model for viability selection. This approach makes it possible to carry out simulation studies, to partition the selection into additive and dominant effects and to remove the effects of non-genetic cofactors from the analysis [14,15]. However, this approach raises two issues. Firstly, they assumed that segregation distortion didn't affect the construction of genetic linkage map. In fact, marker segregation distortion is known to affect the estimates for both recombination fractions in pair-wise analysis of markers and the order of the markers on a linkage group [16-18]. As for the genetic parameters, then, Luo et al [14] adopted the Simplex algorithm [19] to search for the solutions at the cost of computational consuming. Under the liability model proposed by Luo et al [14], therefore, in this paper it is necessary to extend the multipoint approach by combining the estimations of the genetic parameters of SDL with the reconstruction of genetic linkage maps. The new method for SDL mapping was implemented via an expectation and maximization (EM) algorithm rather than Simplex procedure. The genetic factors that might affect the estimates of recombination fractions between adjacent markers would be discussed in detail. A series of Monte Carlo simulation experiments together with a working example from the Mapmaker/QTL software were carried out to verify our approach.

## Methods

### Genetic model

Considering an SDL in an F_2 _population derived from a cross between two inbred lines, we assumed three genotypes at this locus, *AA*, *Aa *and *aa*, to have genotypic values $\sqrt{2}$*a *- *d*, *d *and -$\sqrt{2}$*a *- *d*, respectively, with *a *and *d *indicating additive and dominant effects, and an imaginary trait, liability, invisible to the investigators but visible to nature, controlled the viabilities of individuals. It should be noted that the genetic variance in an F_2 _population was *a*^2 ^+ *d*^2 ^rather than $\frac{1}{2}a^{2}+\frac{1}{4}d^{2}$ as usual. The phenotypic value of the *j*th individual was described by the following linear model,

*z*_*j *_= *g*_*j *_+ *e*_*j*_

where *g*_*j *_was the genotypic value for the *j*th individual, and *ε*_*j *_a normally distributed residual variable with mean zero and standard deviation 1.0, which accounted for polygenes that were linked to the markers and for environmental variation [14,18]. Provided that the liability was subject to natural selection, an individual would survive if *z*_*j *_≥ 0 and would be eliminated from the population if *z*_*j *_< 0. Since all the sampled individuals had survived from the viability selection, the liability of each genotype followed a truncated distribution with a cumulative probability, *G*_*j *_= *h *(*h *= 1, 2, 3), with

$$f_{h}=\mathrm{Pr}(z_{j}\geq 0|G_{j}=h)=\Phi [(2-h)\sqrt{2}a+{\left(-1\right)}^{h}d]$$

where *h *indexed the genotypes of the SDL, and *f*_*h *_was referred to as the relative fitness of the *h*th genotype [14]. The expected frequencies of the three genotypes were

$$p_{AA}=\frac{0.25f_{1}}{0.25f_{1}+0.5f_{2}+0.25f_{3}}=\frac{f_{1}}{f_{1}+2f_{2}+f_{3}}$$

similarly

$$\begin{array}{cc} p_{Aa}=\frac{2f_{2}}{f_{1}+2f_{2}+f_{3}} & p_{aa}=\frac{f_{3}}{f_{1}+2f_{2}+f_{3}} \end{array}$$

### Mapping SDL under a liability model

We assumed that there was no crossing-over interference among the markers on the linkage group considered, an SDL caused segregation distortion of some or all markers linked to the SDL, and three genotypes for each marker had different viability coefficients. Let the order of the *m *markers on a same chromosome be *M*_1_, *M*_2_,...,*M*_*m*_; *x*_*k *_be a dummy variable defined as *x*_*k *_= 1, 0, -1 for a homozygote of P_1_, a heterozygote and a homozygote of P_2 _at the *k*th marker, respectively; *z*_*k *_be indicator for phenotype of the *k*th marker (*M*_*k*_); *r*_*k *_(or *r*_*k*,*k*+1_) be the recombination fraction between the *k*th and (*k*+1)th markers; and *s*_*k*,1 _and *s*_*k*,2 _(0 ≤ *s*_*k*,1 _< +∞ and 0 ≤ *s*_*k*,2 _< +∞ for *k *= 1, 2,...,*m*) be the viability coefficients of *M*_*k*_*m*_*k *_and *m*_*k*_*m*_*k *_relative to *M*_*k*_*M*_*k *_at the *k*th marker.

Now let an SDL locate between the *k*th and (*k*+1)th markers, and *φ*_*jh *_be the indicator function, taking the value of 1, if the *j*th individual belonged to the *h*th possible genotype in the F_2 _population, otherwise taking the value of zero. The parameters were Ω = (*p*_*AA*_, *p*_*Aa*_, *p*_*aa*_, *δ*) or Ω = (*a*, *d*, *δ*), with *δ *indicating the SDL location. The distribution of *φ*_*jh *_was described as

$$\begin{array}{cc} \mathrm{Pr}(\varphi_{jh}|\Omega )={\left(p_{AA}\right)}^{\varphi_{j1}}{\left(p_{Aa}\right)}^{\varphi_{j2}}{\left(p_{aa}\right)}^{\varphi_{j3}}=\frac{f_{1}^{\varphi_{j_{1}}}{\left(2f_{2}\right)}^{\varphi_{j_{2}}}f_{3}^{\varphi_{j_{3}}}}{f_{1}+2f_{2}+f_{3}} & \left(j=1,\cdots ,n\right) \end{array}$$

where *n *was sample size. The likelihood defined with matrix notation was

$$L(\Omega )=\prod_{j=1}^{n}\left\{[{H^{\prime }}_{j}(r_{k,k^{\prime }})\prod_{o=k-1}^{1}{H^{\prime }}_{j}(r_{o,o+1})q_{1}{]}^{\prime }[H_{j}(r_{k^{\prime },k+1})\prod_{o=k+1}^{m-1}H_{j}(r_{o,o+1})c]\frac{f_{1}^{\varphi_{j_{1}}}{\left(2f_{2}\right)}^{\varphi_{j_{2}}}f_{3}^{\varphi_{j_{3}}}}{f_{1}+2f_{2}+f_{3}}\right\}$$

where ${q^{\prime }}_{1}$ = [Pr(*x*_1 _= 1), Pr(*x*_1 _= 0), Pr(*x*_1 _= -1)], *c' *= [1, 1, 1], *' *denoted transpose of a matrix or vector, and the transition probability matrix *H*_*j *_(*r*_*k*,*k*+1_) from marker *M*_*k *_to *M*_*k*+1 _for the *j*th individual was

$$\left[\begin{array}{ccc} \frac{{\left(1-r_{k}\right)}^{2}}{{\left(1-r_{k}\right)}^{2}+2s_{k+1,1}r_{k}(1-r_{k})+s_{k+1,2}r_{k}^{2}} & \frac{2s_{k+1,1}r_{k}(1-r_{k})}{{\left(1-r_{k}\right)}^{2}+2s_{k+1,1}r_{k}(1-r_{k})+s_{k+1,2}r_{k}^{2}} & \frac{s_{k+1,2}r_{k}^{2}}{{\left(1-r_{k}\right)}^{2}+2s_{k+1,1}r_{k}(1-r_{k})+s_{k+1,2}r_{k}^{2}}\\ \frac{r_{k}(1-r_{k})}{\left(1+s_{k+1,2})r_{k}(1-r_{k})+s_{k+1,1}(1-2r_{k}+2r_{k}^{2}\right)} & \frac{s_{k+1,1}(1-2r_{k}+2r_{k}^{2})}{\left(1+s_{k+1,2})r_{k}(1-r_{k})+s_{k+1,1}(1-2r_{k}+2r_{k}^{2}\right)} & \frac{s_{k+1,2}r_{k}(1-r_{k})}{\left(1+s_{k+1,2})r_{k}(1-r_{k})+s_{k+1,1}(1-2r_{k}+2r_{k}^{2}\right)}\\ \frac{r_{k}^{2}}{r_{k}^{2}+2s_{k+1,1}r_{k}(1-r_{k})+s_{k+1,2}{\left(1-r_{k}\right)}^{2}} & \frac{2s_{k+1,1}r_{k}(1-r_{k})}{r_{k}^{2}+2s_{k+1,1}r_{k}(1-r_{k})+s_{k+1,2}{\left(1-r_{k}\right)}^{2}} & \frac{s_{k+1,2}{\left(1-r_{k}\right)}^{2}}{r_{k}^{2}+2s_{k+1,1}r_{k}(1-r_{k})+s_{k+1,2}{\left(1-r_{k}\right)}^{2}} \end{array}\right]$$

There were several ways to find the ML estimates (MLEs) of model parameters. We here adopted an EM algorithm [20] and treated *φ*_*jh *_as missing data. We regarded *δ *as constant for the moment, now the parameter set was *θ *= (*a*, *d*)'. For the EM algorithm, we needed to obtain the expectation of the complete data log-likelihood function,

$$L=C+\sum_{j=1}^{n}\left[p(\varphi_{j1}=1)\ln f_{1}+p(\varphi_{j2}=1)\ln(2f_{2})+p(\varphi_{j3}=1)\ln f_{3}-\ln(f_{1}+2f_{2}+f_{3})\right]$$

where the constant *C *didn't depend on the parameters of interest, and but did depend on the viability coefficients and map distance between adjacent markers, which could be determined by Zhu et al [18]. The EM algorithm was described as follows.

#### E-step

Provided the initial values for the model parameters, i.e., *a*^(0) ^= 0.0 and *d*^(0) ^= 0.0. The posterior probabilities of *φ*_*jh *_= 1 were

$$p(\varphi_{jh}=1)=\frac{\mathrm{Pr}(\varphi_{jh}=1|z_{j_{1}},...,z_{j_{M}})p_{jh}^{\left(0\right)}}{\sum_{o=1}^{3}\mathrm{Pr}(\varphi_{jo}=1|z_{j1},...,z_{jM})p_{jo}^{\left(0\right)}}$$

where $p_{jh}^{\left(0\right)}$ (*h *= 1, 2, 3) was calculated from equation (3), and Pr(*φ*_*jh *_= 1|*z*_*j*1_,...,*z*_*jM*_) (*j *= 1, ...,*n*; *h *= 1, 2, 3) the prior probability of the *h*th genotype of SDL for the *j*th individual conditional on marker information (*z*_*j*_1__,...,*z*_*j_M_*_) by means of the multipoint method [21].

#### M-step

The MLEs of parameters were obtained by the Fisher-scoring algorithm as it was impossible to get their explicit solutions [22]. The *θ *could be updated by

*θ*^(1) ^= *θ*^(0) ^+ **I**^-1^*S*(*θ*^(0)^)

where *S*(*θ*^(0)^) was the score function, and **I **was the Fisher information matrix (more details were given in Appendix). And *θ*^(1) ^would replace *θ*^(0) ^in all subsequent estimating steps, and the procedure was iterated until the convergence occurred. The converged *θ *^(1) ^was the MLEs of *θ *in this M-step.

The *E *and *M *steps were iterated until the convergence occurred.

The MLE for the SDL position could be obtained by examining the likelihood-ratio profile along the chromosome as was commonly done in interval mapping of QTL [9].

Following parameter estimation, we tested an overall null hypothesis that was no effect of SDL at the locus of interest (*δ*). The null hypothesis was formulated as *H*_0_: *a *= *d *= 0.0, which was tested using the likelihood-ratio (*LR*) test statistic:

LR = -2[ln*L*(0, 0, *δ*) - ln*L*(*a*, *d*, *δ*)]

Under the null hypothesis, the statistic LR approximately followed chi-square distribution with two degrees of freedom.

The critical value for power calculation was determined by computing 1,000 permutations [23], the experiment-wise type I error was set at 5%, and the confidence interval of an SDL location was determined by the bootstrapping method [24].

### Simulation model

We simulated one chromosome of 100 cM (or 50 cM) long covered by *m *evenly spaced codominant markers (*m *= 6, 11 or 21) and put a single SDL at position 25 cM (another SDL was put at position 75 cM if necessary). The dominance ratio of the SDL was denoted by *dr *= *d/a*. Given the broad heritability (*h*^2^) and *dr*, the additive and dominant effects could be obtained using numerical algorithm [25]. Based on the method described in Luo *et al.*[14], all genotypes of both distorted markers and SDL for each individual in an F_2 _population were simulated. All simulations were replicated 100 or 1000 times depending on the purpose of the analyses. Empirical power was calculated by counting the number of runs in which test statistics were greater than the critical values [26].

## Results

### Effects of various factors on SDL mapping

In this simulated experiments, the effects of sample size, SDL heritability and marker interval length on SDL mapping were studied, respectively. The performance of the proposed method was evaluated by statistical power, average and standard deviation of estimates with 100 replicates. All parameters and results were listed in Table 1. The results showed the general behavior of QTL mapping, i.e., the estimate for each parameter was very close to its corresponding true value, the power and the precision for SDL mapping increased with the increase in sample size and SDL heritability, respectively. However, marker interval length had slight effect on the power under the three levels studied.

**Table 1:** Results of segregation distortion locus (SDL) mapping under the fitness and liabilty models (100 replications)

Sample size	Interval length (cM)	Broad heritability	Dominance ratio	Power (%)	Positions (cM)	SDL effects		Frequencies of genotypes		
							
						Additive	Dominant	$\hat{p}$_*AA*_	$\hat{p}$_*Aa*_	$\hat{p}$_*aa*_
	True value			/	25.00	0.2124	0.1062	0.2880	0.5412	0.1708
100	10	0.05	0.50	24	42.16(30.55)	0.2011(0.1094)	0.1243(0.1101)	0.2809(0.0716)	0.5485(0.0807)	0.1706(0.0551)
200	10	0.05	0.50	55	30.06(19.33)	0.2143(0.0988)	0.1134(0.0994)	0.2871(0.0499)	0.5440(0.0573)	0.1689(0.0386)
300	10	0.05	0.50	63	28.48(15.68)	0.2097(0.0791)	0.1068(0.0804)	0.2851(0.0345)	0.5454(0.0372)	0.1695(0.0262)
	True value			/	25.00	0.1679	0.1679	0.2634	0.5656	0.1710
200	10	0.05	1.00	53	29.2(21.58)	0.1664(0.0991)	0.1701(0.1007)	0.2672(0.0434)	0.5649(0.0557)	0.1679(0.0386)
	True value			/	25.00	0.2476	0.2476	0.2689	0.5944	0.1367
200	10	0.10	1.00	90	24.44(7.75)	0.2584(0.0916)	0.2681(0.0922)	0.2676(0.0372)	0.6017(0.0405)	0.1307(0.0251)
	True value			/	25.00	0.3162	0.3162	0.2729	0.6170	0.1101
200	10	0.15	1.00	99	25.47(6.66)	0.3066(0.0861)	0.3111(0.0873)	0.2711(0.0349)	0.6181(0.0400)	0.1108(0.0274)
	True value			/	25.00	0.3132	0.1566	0.3046	0.5590	0.1364
200	20	0.15	0.50	92	27.17(13.32)	0.3270(0.0984)	0.1634(0.1023)	0.3007(0.0393)	0.5723(0.0432)	0.1270(0.0260)
200	10	0.15	0.50	91	26.85(12.25)	0.3248(0.0961)	0.1642(0.0987)	0.3086(0.0364)	0.5615(0.0442)	0.1300(0.0285)
200	5	0.15	0.50	93	25.81(10.62)	0.3127(0.0923)	0.1603(0.0911)	0.3037(0.0375)	0.5674(0.0450)	0.1289(0.0263)

The standard deviations are in parentheses. The same is true for the later Tables.

### Mapping multiple SDL

Similar to the interval mapping procedure of Lander and Botstein [9], the single-locus model for SDL mapping was used to search for multiple loci. Eleven markers were evenly placed on a simulated chromosome of length 100 cM. Two SDL each with a 0.5 dominance-ratio and a 0.15 heritability were respectively located at positions 25 cM and 75 cM on the simulated chromosome. One hundred independent simulation runs were performed for a sample size of 200. The results were listed in Table 2. Both loci were identified at almost 100% power. The results from simulation experiments demonstrated that the new method based on single-SDL model may be considered as an approximate approach to search for multiple loci if the SDL are sufficiently separated by markers.

**Table 2:** Results of two segregation distortion loci (SDL) mapping under the fitness and liability models (100 replicates and 200 individuals)

	SDL	Power (%)	Position (cM)	$\hat{a}$	$\hat{d}$	$\hat{p}$_*AA*_	$\hat{p}$_*Aa*_	$\hat{p}$_*aa*_
1	True value	/	25.00	0.3996	0.1998	0.3176	0.5726	0.1098
	Estimate	96	29.13(8.96)	0.4014(0.1097)	0.2018(0.1184)	0.3289(0.0376)	0.5653(0.0425)	0.1058(0.0268)
2	True value	/	75.00	0.3996	0.1998	0.3176	0.5726	0.1098
	Estimate	94	66.75(11.30)	0.4037(0.1064)	0.2053(0.1031)	0.3275(0.0411)	0.5544(0.0450)	0.1181(0.0267)

### A working example

As a demonstration of the proposed method in this paper, we re-analyzed a sample dataset (the source filename: sample.raw) in the MAPMAKER/QTL software [27]. It consisted of 333 F_2 _individuals from a cross between two inbred lines in tomato. Each plant was genotyped for 12 marker loci that were divided into two linkage groups. Single-marker chi-square test showed that 5 and 2 markers on the first and second linkage groups deviated from Mendelian segregation ratios, respectively (data not shown). Given the reconstructed linkage maps using the method of Zhu *et al.*[18], 1000 simulated datasets without segregation distortion were simulated and used to determine the critical value [23]. The confidence interval of a SDL location was determined by the Bootstrap method [24].

**Figure 1:**
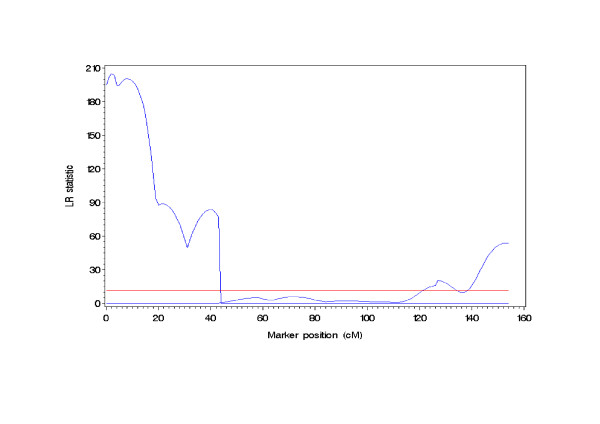
**The likelihood-ratio (LR) score profile for segregation distortion loci mapping against the tomato genome**. The tomato genome derived from Mapmaker 3.0 software (Lander et al. 1987) was composed of two linkage groups.

The map distances between consecutive markers were calculated twice with and without considering SDL. The former was corrected map distance obtained from the method of Zhu *et al.* (2007) [18]; and the latter was uncorrected one using the Mapmaker/EXE 3.0 software [27]. The results were listed in Table 3. The results showed that the corrected map distances differed from the uncorrected ones when there were distorted markers. The genetic reason of these inconsistencies would be discussed in the following section. Using the proposed method here, a total of four SDL were mapped (Table 4, Fig 1). Two SDL were on the first linkage group and the others on the second one. The genetic parameters for the four SDL were listed in Table 4. The results showed that the distortion was stronger for the first linkage group than for the second one (Fig 1). It resulted in a maximum difference between the corrected and uncorrected map distances for the first marker interval on the first linkage group. Moreover, two linked SDL on the second linkage group also gave rise to two big differences (Table 3). As compared to a single SDL, therefore, linked SDL had a larger effect on the estimate of map distance.

**Table 3:** The uncorrected and corrected map distances in the real data analysis

	Linkage group 1				Linkage group 2					
		
Map distance	1	2	3	4	1	2	3	4	5	6
Corrected	5.29	14.93	11.60	12.77	14.58	6.24	18.88	24.04	17.13	27.69
Uncorrected	4.18	15.01	11.88	12.19	14.76	6.38	18.88	24.04	18.12	28.69

Uncorrected and corrected map distances were calculated by Mapmaker/EXE 3.0 software (Lander et al 1987) and DistortedMap software (Zhu et al 2007), respectively. The same is true for Tables 5 and 6.

**Table 4:** Results of segregation distortion loci (SDL) mapping in a real data analysis

Linkage group	Position (cM)	Confidence interval (95%)	Nearest marker to SDL	$\hat{a}$	$\hat{d}$	Dominance ratio	$\hat{p}$_*AA*_	$\hat{p}$_*Aa*_	$\hat{p}$_*aa*_	Selection types
1	2	1~11	T175	1.4628	-0.1669	-0.11	0.5249	0.4598	0.0153	Zygotic
	41	27~44	T508	0.6792	0.0874	0.13	0.3992	0.8280	0.0728	Zygotic
2	84	57~90	T209	-0.0164	0.3133	-19.10	0.1842	0.6229	0.1929	Zygotic
	109	85~127	T17	0.4969	-0.3184	-0.64	0.4347	0.3853	0.1800	Zygotic

### Effect of genetic model of SDL on the estimation of map distance

In this section, our purpose was to make clear the genetic reason for the inconsistencies between corrected and uncorrected map distances when there were distorted markers. Six evenly spaced codominant markers were simulated on a single-chromosome segment of length 50 cM. Two linked SDL with locations at positions 10 and 20 cM (exactly the 2nd and 3rd marker loci) were simulated on the simulated chromosome. One hundred simulation runs were performed for a sample size of 300. Each of datasets was analyzed twice by the method of Zhu *et al.* (2007) [18] and the Mapmaker/EXE 3.0 software [27]. The former was corrected map distance and the latter uncorrected one. For an additive-dominant model, all genetic parameters and the results were listed in Table 5. Results showed that uncorrected map distance was underestimated for most cases, overestimated for opposite dominant effects, and unbiased for all negative additive effects. The results from the real dataset analysis above partly confirmed the result that opposite dominant effects of the two linked SDL on the second linkage group (Table 4) gave rise to the overestimation (Table 3). For an epistatic model, all genetic parameters and the results were listed in Table 6. Results showed that uncorrected map distance was underestimated for most situations, overestimated for negative additive-by-additive or negative dominant-by-dominant effects, and unbiased for additive-by-dominant effect. As we expected, corrected genetic distances were unbiased when considering SDL (Table 5 and 6). Hence, corrected linkage maps were recommended to be used for further QTL or SDL analysis unless there was strong evidence to believe that all markers presented typical Mendelian segregation.

**Table 5:** Effect of genetic modes of two linked SDL on the estimates of map distances under the additive-dominant model

					Estimates of map distances (cM)				
					
*a*_1_	*d*_1_	*a*_2_	*d*_2_	Method	The 1st interval	The 2nd interval	The 3rd interval	The 4th interval	The 5th interval
0.5	0.0	0.5	0.0	Corrected	10.13(1.25)	9.88(1.02)	10.22(1.51)	10.06(1.50)	10.27(1.53)
				Uncorrected	9.99(1.43)	7.27(1.38)	10.00(1.52)	9.91(1.48)	10.16(1.52)
0.5	0.0	-0.5	0.0	Corrected	9.92(1.41)	9.89(1.21)	9.92(1.52)	10.08(1.51)	10.01(1.59)
				Uncorrected	10.01(1.43)	7.20(1.22)	10.01(1.53)	10.17(1.51)	10.07(1.59)
-0.5	0.0	-0.5	0.0	Corrected	10.32(1.58)	10.48(1.53)	9.96(1.33)	10.01(1.75)	10.09(1.59)
				Uncorrected	10.41(1.57)	10.65(1.55)	10.11(1.35)	10.07(1.75)	10.10(1.59)
0.0	0.5	0.0	0.5	Corrected	9.99(1.60)	10.18(1.12)	10.04(1.42)	9.97(1.51)	9.98(1.51)
				Uncorrected	10.03(1.60)	6.28(1.13)	10.08(1.44)	10.02(1.53)	10.01(1.51)
0.0	0.5	0.0	-0.5	Corrected	10.16(1.62)	10.05(1.27)	10.09(1.49)	9.81(1.47)	10.24(1.36)
				Uncorrected	10.16(1.72)	15.64(1.97)	10.09(1.59)	9.81(1.48)	10.24(1.36)
0.0	-0.5	0.0	-0.5	Corrected	10.14(1.33)	9.94(0.95)	10.38(1.50)	10.08(1.38)	10.09(1.63)
				Uncorrected	9.98(1.53)	6.58(1.17)	10.02(1.56)	9.92(1.34)	10.06(1.62)

*a*_1 _and *a*_2 _(*d*_1 _and *d*_2_) are the additive (dominant) effects of the two SDL.

**Table 6:** Effect of genetic modes of two linked SDL on the estimates of map distances under the epistatic genetic model

					Estimates of map distances (cM)				
					
*i*_11_	*i*_12_	*i*_21_	*i*_22_	Method	The 1st interval	The 2nd interval	The 3rd interval	The 4th interval	The 5th interval
0.5	0.0	0.0	0.0	Corrected	9.91(1.40)	9.94(1.35)	10.10(1.59)	9.90(1.50)	9.98(1.33)
				Uncorrected	9.91(1.40)	9.20(1.35)	10.10(1.59)	9.90(1.50)	9.98(1.33)
-0.5	0.0	0.0	0.0	Corrected	9.74(1.60)	10.40(1.20)	10.18(1.39)	9.48(1.45)	10.10(1.31)
				Uncorrected	9.86(1.62)	14.64(2.02)	10.33(1.50)	9.61(1.46)	10.16(1.32)
0.0	0.5	0.0	0.0	Corrected	10.13(1.39)	9.97(1.37)	10.01(1.66)	10.08(1.57)	10.21(1.69)
				Uncorrected	10.17(1.39)	9.95(1.37)	10.05(1.67)	10.09(1.57)	10.21(1.69)
0.0	-0.5	0.0	0.0	Corrected	10.20(1.56)	10.05(1.52)	10.20(1.52)	10.05(1.58)	10.18(1.59)
				Uncorrected	10.21(1.57)	10.05(1.53)	10.19(1.53)	10.03(1.59)	10.18(1.59)
0.0	0.0	0.0	0.5	Corrected	9.98(1.64)	9.24(1.37)	10.14(1.55)	9.73(1.42)	10.08(1.57)
				Uncorrected	10.00(1.64)	7.25(1.38)	10.15(1.55)	9.73(1.43)	10.08(1.57)
0.0	0.0	0.0	-0.5	Corrected	10.02(1.39)	10.09(1.30)	10.02(1.10)	9.97(1.37)	10.23(1.44)
				Uncorrected	10.01(1.59)	12.89(1.90)	10.01(1.30)	9.97(1.47)	10.23(1.44)

*i*_11_, *i*_12 _(*i*_21_) and *i*_22 _are additive × additive, additive × dominant and dominance × dominance epistatic effects of the two SDL, respectively, and the additive and the dominant effects for the two SDL are set up at zero.

## Discussion

For SDL mapping, most researchers concentrate their attention upon detecting and testing either the selection coefficients or the degree of dominance under the fitness model [7,10,11]. Luo *et al.*[14] pioneered in the development of SDL mapping under a liability model. Zhu et al. [18] proposed a new method for the reconstruction of linkage maps with distorted, dominant and missing markers. Under the liability model, we developed a method to simultaneously estimate the position and the effects of SDL as well as the recombination fractions between adjacent markers. This approach remains the merits of Luo *et al.*[14] but differs from others in several aspects. Firstly, it combines the detection of SDL with the reconstruction of marker linkage map. The position and the effect of SDL can be estimated along with the selection coefficient and the degree of dominance. Then, the proposed method may be used to elucidate the relationship between the viability selection and genetic linkage. Thirdly, the likelihood function is involved in the distribution of genotypes of SDL rather than that of marker genotypes in the previous studies [11,28]. Finally, we adopted an EM algorithm rather than the Simplex procedure to estimate the genetic parameters. Of course, we should notice one common assumption of the mentioned-above approaches that marker segregation distortion is caused by some genetic or viability reasons. For genetic reason, there are two different mechanisms for segregation distortion, one at the gametic level and the other at the zygotic level. In both cases, observable phenotypes are distorted for marker loci in the chromosomal region close to the SDL. Thus the two mechanisms are included in our proposed method. Although we have no way to distinguish them in SDL mapping, the results from the genotype and allele tests [29] for the marker closest to the SDL can be used to infer the presence of zygotic or gametic viability selection in an F_2 _population but not in backcross, double haploid and recombinant inbred line populations. Moreover, it should be noted that genetic linkage between distorted markers has been carefully discussed in Wu *et al.* (2007) [30].

There are two primary routes by which selection can affect the extent of linkage disequilibrium [31]. The first is a hitchhiking effect, in which an entire haplotype that flanks a favored variant can be rapidly swept to high frequency or even fixation [32]. The second way in which selection can affect linkage disequilibrium is through epistatic selection for combination of alleles at two or more loci on the same chromosome [33]. This selection form leads to the association of the particular alleles at different loci. The major difficulty in linkage disequilibrium-based mapping is to quantify the relationship between recombination fraction and linkage disequilibrium measurement. Our analyses are confined to exclude the factors that influence linkage disequilibrium except linkage and selection. We first combine the viability selection with quantitative genetics model, and then explore the relationship between genetic modes of the viability genes and the estimates of the recombination fraction. The simulation studies indicated that most of the genetic modes of the viability genes at the two linked SDL may result in underestimation of genetic distance. We hope that the tentative attempt will make for elucidating the genetic relationship between viability selection and genetic linkage.

In addition, it will be interesting and challenging to combine the SDL analysis with QTL mapping to see what the effects of distorted markers has on the results of QTL mapping. While doing this, one may take a risk of detecting false QTL not due to their genetic effects on the quantitative traits but due to violation of the Mendelian segregation law. It will be a great breakthrough in quantitative genetics area if we can develop a method to separate the effects of viability loci from the effects of QTL [14]. By reason of the complexity of the combined analysis, the related investigations will be discussed separately elsewhere.

## Conclusion

Our results suggested that the proposed method can serve as a powerful alternative to existing methods. Under the liability model, the new method can simultaneously estimate the position and the effects of SDL as well as the recombination fractions between adjacent markers, and also be used to probe into the genetic mechanism for the bias of uncorrected map distance and to elucidate the relationship between the viability selection and genetic linkage.

## Authors' contributions

CZ designed and carried out the simulation study, and drafted the manuscript. YMZ conceived of the study, participated in the design, coordinated it and revised the manuscript. All authors read and approved the final manuscript.

## Appendix: Fisher-scoring algorithms for obtaining MLEs of parameters

The Fisher-scoring algorithm can be used to estimate parameters in the M-step of EM algorithm. Let *θ *= (*a*, *d*)^*T*^. The newly estimated *θ *can be expressed by the score-function vector *S *and the Fisher information matrix **I**,

$$\theta^{\left(1\right)}=\theta^{\left(0\right)}+I_{\theta =\theta^{\left(0\right)}}^{-1}S_{\theta =\theta^{\left(0\right)}}$$

where *S *= ∂ln*L*/∂*θ *= (∂ln*L*/∂*a*, ∂ln*L*/∂*d*)^*T *^is score function, and

$$I=-E\left(\frac{\partial^{2}\ln L}{\partial \theta \partial \eta }\right)=-\left(\begin{array}{cc} E\left(\frac{\partial^{2}\ln L}{\partial a^{2}}\right) & E\left(\frac{\partial^{2}\ln L}{\partial a\partial d}\right)\\ E\left(\frac{\partial^{2}\ln L}{\partial d\partial a}\right) & E\left(\frac{\partial^{2}\ln L}{\partial d^{2}}\right) \end{array}\right)$$

is Fisher information matrix.

More specifically, the score function and the Fisher information index of the expected complete data log-likelihood can be derived using

$$\begin{array}{cc} \frac{\partial \ln L}{\partial \theta } & =\sum_{j=1}^{n}\left[w(\varphi_{j1}=1)\frac{\partial \ln(f_{1})}{\partial \theta }+w(\varphi_{j2}=1)\frac{\partial \ln(2f_{2})}{\partial \theta }+w(\varphi_{j3}=1)\frac{\partial \ln(f_{3})}{\partial \theta }-\frac{\partial \ln(f_{1}+2f_{2}+f_{3})}{\partial \theta }\right]\\ & =\sum_{j=1}^{n}\left[\frac{w(\varphi_{j1}=1)}{f_{1}}\frac{\partial (f_{1})}{\partial \theta }+\frac{w(\varphi_{j2}=1)}{2f_{2}}\frac{\partial (2f_{2})}{\partial \theta }+\frac{w(\varphi_{j3}=1)}{f_{3}}\frac{\partial (f_{3})}{\partial \theta }-\frac{\partial (f_{1}+2f_{2}+f_{3})/\partial \theta }{f_{1}+2f_{2}+f_{3}}\right]\\ & =\sum_{j=1}^{n}\left[\sum_{h=1}^{3}\frac{w(\varphi_{jh}=1)}{f_{j}}\frac{\partial f_{j}}{\partial \theta }-\frac{\partial (f_{1}+2f_{2}+f_{3})/\partial \theta }{f_{1}+2f_{2}+f_{3}}\right] \end{array}$$

Let *μ*_*h *_= $\sqrt{2}$(2 - *h*)*a *+ (-1)^*h*^*d *for *h *= 1, 2, 3

$$\begin{array}{cc} \frac{\partial f_{h}}{\partial \theta } & =-\frac{1}{\sqrt{2\pi }}\int_{-\infty }^{0}\frac{\partial e^{-\frac{{\left(z_{jh}-\mu_{h}\right)}^{2}}{2}}}{\partial \theta }dz_{jh}\\ & =-\frac{1}{\sqrt{2\pi }}\left\{\int_{-\infty }^{0}e^{-\frac{{\left(z_{jh}-\mu_{h}\right)}^{2}}{2}}d\left[-\frac{{\left(z_{jh}-\mu_{h}\right)}^{2}}{2}\right]\right\}\frac{\partial \mu_{h}}{\partial \theta }\\ & =\frac{1}{\sqrt{2\pi }}\exp\left[-\frac{{\left(0-\mu_{h}\right)}^{2}}{2}\right]\frac{\partial \mu_{h}}{\partial \theta }\\ & =\frac{1}{\sqrt{2\pi }}\exp\left[-\frac{\mu_{h}^{2}}{2}\right]\frac{\partial \mu_{h}}{\partial \theta } \end{array}$$

with $\frac{\partial \mu_{h}}{\partial \theta }=\sqrt{2}(2-h)$ or (-1)^*h *^when *θ *= *a *or *d *correspondingly. Hence

$$\frac{\partial \ln L}{\partial a}=[\sum_{j=1}^{n}\sum_{h=1}^{3}\frac{w(\varphi_{jh}=1)}{f_{h}}-\frac{n}{f_{1}+2f_{2}+f_{3}}]\sum_{h=1}^{3}\frac{\left(2-h\right)}{\sqrt{\pi }}e^{-\frac{{\left(-\sqrt{2}(2-h)a-{\left(-1\right)}^{h}d\right)}^{2}}{2}}$$

$$\frac{\partial \ln L}{\partial d}=\sum_{j=1}^{n}\sum_{h=1}^{3}\frac{w(\varphi_{jh}=1){\left(-1\right)}^{h}e^{\frac{{\left(-\sqrt{2}(2-h)a-{\left(-1\right)}^{h}d\right)}^{2}}{2}}}{\sqrt{2\pi }f_{h}}-\frac{n{\left(-1\right)}^{h}[e^{\frac{{\left(-\sqrt{2}a+d\right)}^{2}}{2}}+2e^{\frac{{\left(-d\right)}^{2}}{2}}+e^{\frac{{\left(\sqrt{2}a+d\right)}^{2}}{2}}]}{\sqrt{2\pi }(f_{1}+2f_{2}+f_{3})}$$

The second partial derivatives are more messy but a general form was found as

$$\begin{array}{ll} \frac{\partial^{2}\ln L}{\partial \theta \partial \eta } & =\sum_{j=1}^{n}\{\sum_{h=1}^{3}w(\varphi_{jh}=1)[\frac{1}{f_{h}}\frac{\partial^{2}f_{h}}{\partial \theta \partial \eta }-\frac{1}{f_{h}^{2}}\frac{\partial f_{h}}{\partial \theta }\frac{\partial f_{h}}{\partial \eta }]-\frac{1}{f_{1}+2f_{2}+f_{3}}\frac{\partial^{2}{\left(f_{1}+2f_{2}+f_{3}\right)}^{2}}{\partial \theta \partial \eta }\\ & +\frac{1}{{\left(f_{1}+2f_{2}+f_{3}\right)}^{2}}\frac{\partial (f_{1}+2f_{2}+f_{3})}{\partial \theta }\frac{\partial (f_{1}+2f_{2}+f_{3})}{\partial \eta }\} \end{array}$$

where

$$\begin{array}{cc} \frac{\partial^{2}f_{h}}{\partial \theta \partial \eta } & =\frac{1}{\sqrt{2\pi }}\exp(-\frac{\mu_{h}^{2}}{2})(-\mu_{h})\frac{\partial \mu_{h}}{\partial \theta }\frac{\partial \mu_{h}}{\partial \eta }+\frac{1}{\sqrt{2\pi }}\exp(-\frac{\mu_{h}^{2}}{2})\frac{\partial^{2}\mu_{h}}{\partial \theta \partial \eta }\\ & =\frac{1}{\sqrt{2\pi }}\exp(-\frac{\mu_{h}^{2}}{2})(-\mu_{h})\frac{\partial \mu_{h}}{\partial \theta }\frac{\partial \mu_{h}}{\partial \eta } \end{array}$$
